# AI and automation: democratizing automation and the evolution towards true AI-autonomous robotics

**DOI:** 10.1039/d5sc03183d

**Published:** 2025-08-04

**Authors:** Lauren Takahashi, Mikael Kuwahara, Keisuke Takahashi

**Affiliations:** a Department of Chemistry, Hokkaido University North 10, West 8 Sapporo 060-0810 Japan lauren.takahashi@sci.hokudai.ac.jp keisuke.takahashi@sci.hokudai.ac.jp; b List Sustainable Digital Transformation Catalyst Collaboration Research Platform, Institute for Chemical Reaction Design and Discovery, Hokkaido University Sapporo 001-0021 Japan lauren.takahashi@sci.hokudai.ac.jp

## Abstract

The integration of artificial intelligence (AI) and robotics is fundamentally transforming the landscape of chemical and materials research, enabling the rise of autonomous laboratories capable of conducting high-throughput, data-driven experimentation with minimal human intervention. The current state, challenges, and future directions of AI-driven automation in the laboratory are explored, emphasizing the technical, ethical, and cultural shifts required to support this paradigm. Efforts to democratize access to laboratory automation *via* open-source hardware, modular systems, and digitial fabrication are highlighted, showcasing methods of innovation for smaller research groups that conduct research alongside well-funded institutions. Concurrently, the need for robust safety systems, validation protocols, and interdisciplinary collaboration are stressed to ensure reliability, transparency, and inclusivity. Beyond technical capability, the emergence of AI systems capable of hypothesis generation and scientific reasoning raises critical questions about the evolving nature of creativity, intuition, and authorship in science. Rather than replacing human researchers, an argument is made for a model of collaborative intelligence- where humans and machines co-create knowledge, each contributing distinct strengths. This perspective proposes that the future of research lies not only in smarter tools but in the development of smarter, ethically grounded systems that amplify human insight and redefine the very practice of science in the 21st century.

## Abstract 2

Through artificial intelligence and robotics, autonomous labs are transforming chemical and materials research by enabling high-throughput, data-driven experiments with minimal human input.

## Graphical abstract

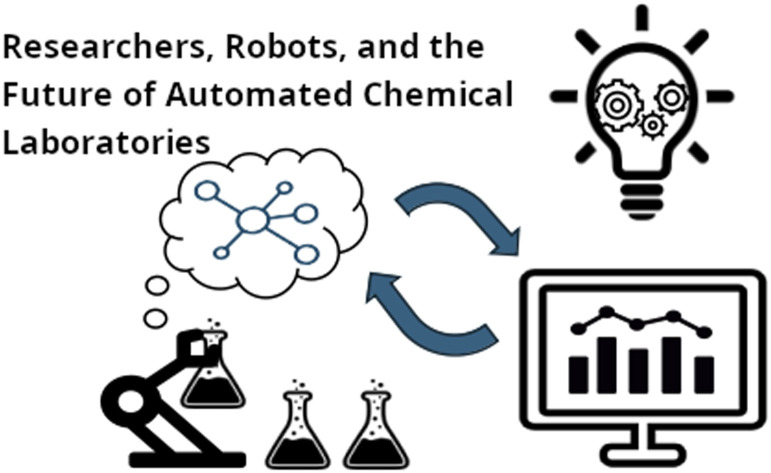

## 1 Introduction: the need for automation and AI in chemistry and materials science

**Fig. 1:**
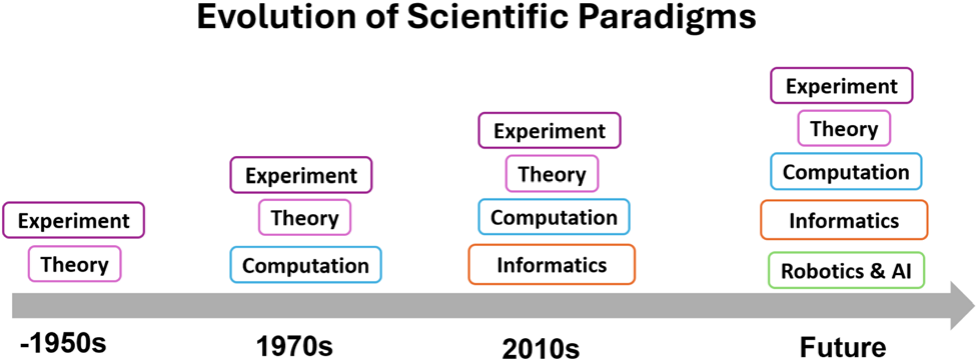
Approaches to chemical research have expanded over the years due to advances in technology and scientific discoveries, expanding from traditional experimental and theoretical approaches to include computational approaches and, thanks to the exponential growth in data availability, informatics approaches. Current developments have progressed towards the birth of a new approach to scientific research, where robotics and artificial intelligence are used together in order to automate laboratory setups and research activities.

Chemistry has experienced an exponential level of growth and development over the past several decades. The advent of the computer in the 20th century has expanded the approaches that can be taken for chemical research, allowing the chemistry field to advance through the interplay of four foundational approaches: experiment, theory, computation, and- in recent years- informatics. The technological advances experienced globally throughout the past hundred years have resulted in an explosion of scientific advancement and exponential growth in scientific discoveries, development of new technologies, and irreversible changes to modern society that have promoted international collaboration, increased knowledge exchange, and improved the availability and accessibility of scientific research. Recent adjacent developments in computer science and the overall technological developments observed globally have resulted in the emergence of a fifth paradigm in chemical research as seen in Fig. 1: the integration of robotics, automation, and artificial intelligence (AI) into chemical research. These technologies are reshaping how experiments are designed, executed, and interpreted, enabling unprecedented levels of throughput, reproducibility, and adaptability. Particularly in cases like materials science and catalysis, this shift is opening new frontiers for autonomous discovery, closed-loop optimization, and data-driven innovation.^1,2^

One major consequence of these developments is the rapid accumulation of chemical data at scales far beyond what individual researchers can manually analyze. With the rise of data science, it has become possible to process and interpret high-dimensional datasets, revealing patterns and correlations that are otherwise inaccessible due to levels of dimensionality that are beyond an individual researcher's physical processing capabilities. This allows machines to learn complex relationships between structure, processing conditions, and performance—accelerating property prediction and guiding experimental design more efficiently.^3^ Machine learning has, for example, been applied towards endeavors involving battery development and C–N cross coupling.^4,5^ Other approaches such as deep learning and neural networks have been used for context-dependent design of induced-fit enzymes and predicting protein structures.^6,7^ Simultaneously, breakthroughs in natural language processing and the establishment of BERT and GPT have expanded the tools available for mining scientific literature, generating hypotheses, and predicting chemical reactions.^8–12^ Models such as ChemBERTa-2, MolFormer, and CrysGNN have been developed in order to process chemical language and other chemistry-related text, molecular structures, and similar data in order to carry out functions such as molecular property prediction, molecule generation, reaction prediction, and material discovery based on graph models of crystal structures.^13–15^ Other models like AtomGPT, ChemDM, and nach0 have been developed to handle multimodal chemical reasoning, generate materials through diffusion-based inverse design, and plan and conduct chemistry workflows.^16–18^ Together, these AI-driven technologies are reshaping how chemists generate, interact with, and act upon knowledge, paving the way for the next generation of autonomous research systems in the chemistry field.

These advances—particularly those at the intersection of informatics, robotics, and AI—have made possible a new vision for chemical research: the autonomous laboratory. Autonomous robotic labs offer significant advantages and are highly appealing as they provide abilities such as conducting thousands of experiments daily, accelerating material discovery, and improving synthetic chemistry automation. By taking over repetitive, time-consuming, or hazardous tasks, these systems improve researcher safety and free up human creativity for higher-level scientific reasoning while also improving efficiency and reducing human error. Unsurprisingly, the appeal of autonomous labs has grown rapidly across academia and industry, driven by the promise of faster research cycles and more efficient research and development pipelines.^19–21^

The growing prominence of automation and robotics raises questions about the nature of research, requiring a more critical examination of their roles and influences. While artificial intelligence excels at pattern recognition and predictions through the use of machine learning, deep learning, feature extraction, analysis, and classification, it still lacks capacities that define human scientific inquiry—including causal reasoning, mechanistic understanding, creativity, and the ability to form and refine hypotheses. This has become clearer as reports begin to note the limitations of AI Scientists in areas that require complex reasoning, cross-system coordination, and post-experiment evaluation and validation: all tasks that are core components of scientific research.^22,23^ Future advances must therefore go beyond incremental improvements in machine learning and move toward systems capable of autonomous scientific decision-making—not only in execution, but also in conceptualization, interpretation, and innovation. Moreover, the rise of robot-centered laboratories may require a fundamental rethinking of how scientific environments are designed and how researchers interact with them. As autonomous systems continue to reshape the research landscape, they not only offer new capabilities but also challenge traditional paradigms of experimentation, interpretation, and collaboration.

Advances in informatics and concurrent developments in robotics have jumpstarted a shift in the human–robot dynamics in research practices, which will likely have a great impact not only on current research practices but also on the shape and nature of chemical research in the future. This perspective explores the current state and barriers faced regarding automation, paths towards more accessible and affordable robotics, the impact LLMs have had on society and the challenges faced by language generation, and the ways in which these technologies will influence our research environments. Finally, this perspective explores how our relationships with these technologies may change and evolve over time, and the impact of human–robot collaboration will have on future research practices and the nature of chemical research.

## 2 Automation in chemical and materials research: current advances and barriers

Automation in the chemical and materials research sphere is highly attractive on several fronts. One of its benefits is its ability to conduct mundane and repetitive lab work. Common tasks well-suited for automation include weighing and mixing reagents, running parallel syntheses under varying conditions, conducting routine characterization measurements, cleaning glassware, and managing data logging and sample tracking. Automation has been successfully used for proteomics sample preparation, for example, with reports of great improvements in efficiency and reductions in processing costs.^24^ Additional applications have also found success in titration acivities as well as electrochemical research handling electrode cells and motion control.^25–27^ Areas such as electrochemistry benefit as well, as automation and robotics can be used to carry out research in air-free environments or other sensitive conditions.^28^ Employment of robots in a research environment also allows for robots to undertake experiments in hazardous conditions or handle toxic and dangerous chemicals, improving the safety of the human researchers. These qualities are highly attractive to researchers, as they help free up time and resources previously spent on routine lab work.^29^

By offloading repetitive, labor-intensive tasks to automated systems, researchers can shift their focus to more cutting-edge, complex, and creative challenges—such as designing novel experiments, interpreting complex results, and developing new hypotheses. Automation also contributes to safer laboratory environments and reduces common errors associated with manual procedures. This evolution mirrors earlier technological shifts in science: before the advent of calculators and computers, scientists devoted significant effort to manual calculation. With those tools, the focus naturally shifted from how to calculate to what to calculate—enabling deeper exploration and higher-level thinking. In the same way, laboratory automation allows researchers to redirect their cognitive efforts toward innovation and discovery, rather than procedural execution.

**Fig. 2:**
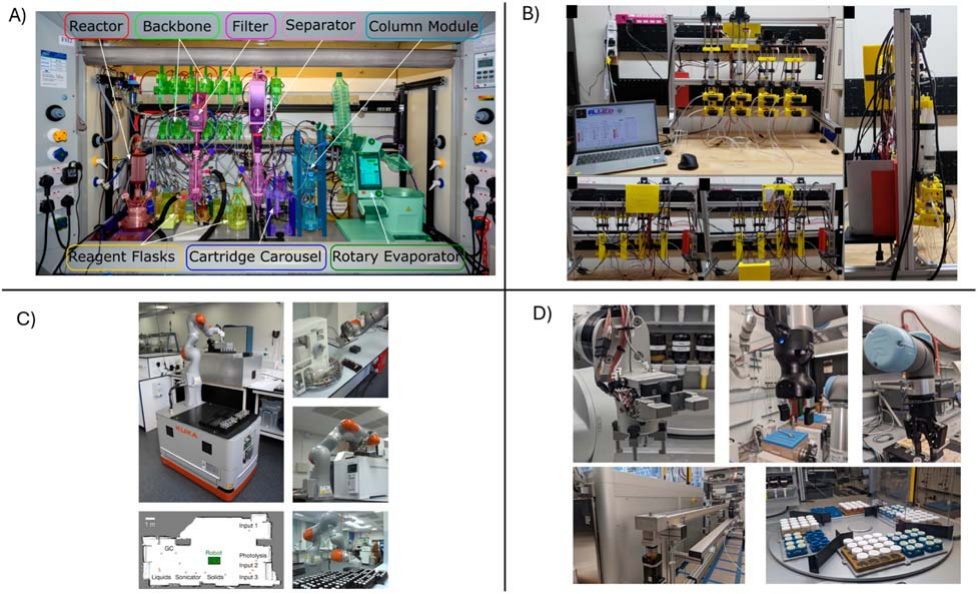
Examples of robotics and automation developed and adopted in chemistry laboratories. (A) Modular, universal ChemPU platform from ref. 40 with permission. (B) FLUID Reprinted with permission from ref. 33. Copyright 2025 American Chemical Society. (C) a Kuka mobile robot platform and its operating environment. Reprinted with permission from ref. 35. (D) UR5e robotic arms used for sample preparation, retrieval, and characterization. Reprinted with permission from ref. 36.

There has been a growing movement toward integrating automation into chemical laboratories. Fig. 2 illustrates several examples of how robotics and automation are incorporated into chemical research. One notable example is the Chemputer (Fig. 2a), a modular robotic system designed for automated chemical synthesis execution, controlled by a custom software platform.^30^ The Chemputer integrates three major components—hardware modules, software, and a control system using the Chemical Description Language (XDL)— to autonomously perform complex synthetic workflows.^19,31,32^ Its modularity allows researchers to customize reaction setups, insert manual inputs when needed, and maintain full control over experimental design, while also improving reproducibility, data integrity, and efficiency by minimizing human error. Open source resources and 3D printing technologies have also been used to develop robots like FLUID (Fig. 2b) for material synthesis.^33^ Other reconfigurable platforms for automated chemical synthesis have been developed to optimize a wide range of reactions.^34^ For example, mobile robots such as the Kuka mobile robot found in Fig. 2c have been designed to handle vials, operate a variety of experimental instruments, and disperse materials with high accuracy over long periods of time.^35^ Robotic arms have also drawn attention, where arms like the UR5e arms illustrated in Fig. 2d are used to prepare samples, load and unload racks to and from box furnaces, and retrieve and characterize samples.^36^

These systems accelerate reaction screening and synthesis workflows, freeing researchers to focus on more intellectually demanding tasks such as experimental design, data interpretation, and hypothesis generation. Automation is also becoming increasingly prevalent in the chemical industry, where companies like AstraZeneca and startups such as Emerald Cloud Lab are integrating robotics, AI, and cloud infrastructure to build fully automated laboratories.^37–39^ These systems enable remote experimentation, streamline drug discovery and synthesis, and offer scalable solutions for optimizing chemical processes—reshaping the way research is conducted across both academia and industry.

Automated systems such as these offer significant potential for the future of chemical research. In fact, there is already movement towards incorporating automation into the chemistry space.^35,41–43^ With parallel advancements in remote laboratory infrastructure, it is increasingly feasible to envision robot-only laboratories that operate independently of direct human intervention. However, despite the benefits and opportunities provided by automation, there are several barriers that hinder its widespread adoption and utilization.

Chief among these is the high cost of implementation. Establishing an autonomous laboratory demands a substantial financial investment, requiring high-end robotics, integrated AI models, high-throughput instrumentation, and specialized infrastructure. Even after setup, ongoing expenses for maintenance, repairs, and upgrades further add to the financial burden of running an autonomous laboratory. These costs are compounded by the price of reagents and materials used in large-scale or continuous experimentation—which, depending on the chemical involved, can be prohibitively expensive. Commercial platforms such as RoboRXN and AstraZeneca's AI lab, while powerful and cutting-edge, often require investments in the range of millions of dollars. This level of funding is typically out of reach for small- and mid-sized research groups or companies, effectively restricting access to well-funded institutions and industry players. As a result, the current landscape risks creating a divide between laboratories that can afford full automation and those that cannot—potentially reinforcing disparities in research capabilities. Moving forward, the development of more modular, cost-effective, and open-source automation tools may help democratize access and broaden the impact of these transformative technologies.

In addition to the high financial costs associated with adopting robotics and establishing autonomous laboratories, there are also significant concerns regarding the flexibility and adaptability of these technologies. The strength of autonomous robotic labs lies in their ability to efficiently perform routine, pre-designed experiments at scale—but this often comes at the expense of versatility when faced with unpredictable or novel scientific challenges. These systems are not yet equipped to tackle tasks that require creativity, intuition, or interpretive reasoning—qualities that lie at the heart of hypothesis-driven research. Such human traits are cultivated through years of education, observation, and accumulated experience, each shaped by the individual researcher's unique background and intellectual journey. In contrast, robots lack this contextual infrastructure. They can only operate within the scope of their programming or training data, and often fail when presented with scenarios outside those bounds. One promising direction is to develop systems that can learn not only from data, but from the behavior of expert researchers—including the tacit knowledge that is often difficult to document. For example, chemists may infer material activity from subtle, subjective cues like slight changes in color, viscosity, or texture—observations that may not be explicitly written in lab notebooks, but are deeply meaningful in practice. Mimicking such expert intuition would require capturing the nuanced, often unspoken decision-making processes in the lab and translating them into machine-understandable formats, perhaps through vision systems, multimodal learning, or reinforcement learning based on expert demonstrations.

While robotics and automation are rapidly becoming central to materials synthesis, reaction engineering, and characterization, most current systems remain highly specialized—designed for specific reactions, chemical types, or environmental conditions. This narrow focus, along with the high costs of commercial platforms, has limited adoption to well-funded institutions and industries. To overcome these barriers, open-source and modular approaches are gaining traction.^44,45^ Sharing hardware blueprints and software openly allows researchers to customize and adapt robotic platforms to their specific experimental needs, fostering a more inclusive and collaborative ecosystem. In parallel, low-cost tools such as single-board microcontrollers (*e.g.*, Arduino or Raspberry Pi) and affordable actuators are making it possible to build flexible automation systems without large budgets. One particularly promising development is the use of 3D printing to create custom lab tools, parts, and components for robotic systems. This approach dramatically reduces the cost and time needed for prototyping, while also enabling the rapid iteration of highly tailored solutions. From custom reaction vessels to robotic grippers and sensor mounts, 3D printing empowers researchers to innovate locally and adapt tools on demand.^46,47^ An example of this approach is FLUID, an open-source, 3D-printed robotic platform developed specifically for materials synthesis.^33^ FLUID offers a fully accessible blueprint for both hardware and software, enabling researchers to build customizable automation systems using affordable, readily available tools. By lowering the technical and financial barriers traditionally associated with laboratory automation, FLUID represents a key step toward democratizing materials innovation. In a similar manner, open source has also been utilized in order to design automated systems like FINDUS; a platform that automates pipette-related tasks; OTTO, a system designed to handle liquids for automating prep processes; and the Jubilee workstation, system that are designed for closed-loop experimentation while preserving transparency and customization.^48–50^ Together, these low-cost and open-source technologies are helping to democratize laboratory automation, making advanced experimentation more accessible, customizable, and scalable across research communities worldwide. As this trend continues, the future of automated research may no longer be limited by funding, but instead shaped by creativity, collaboration, and shared innovation.

## 3 AI in chemistry: beyond machine learning and large language models

**Fig. 3:**
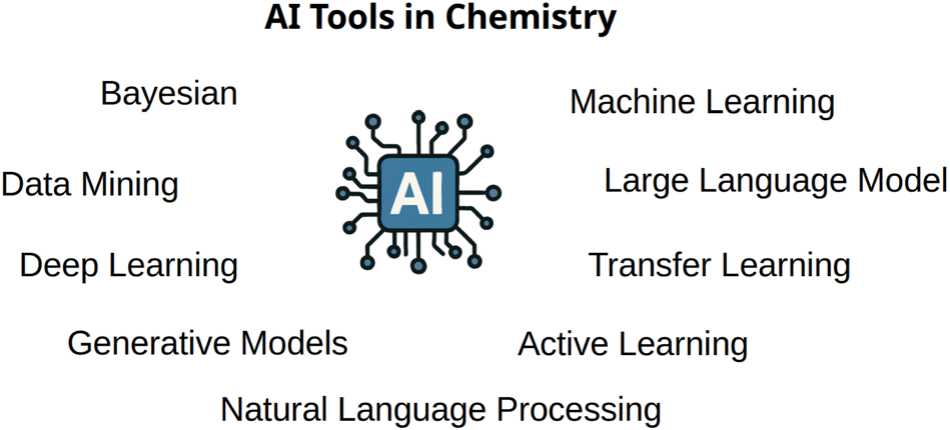
Artifical intelligence tools used in chemistry range from natural language processing models, BERTs and GPTs, systems such as AlphaFold, and machine learning tools and algorithms.

Artificial intelligence has quickly established a strong foothold in modern scientific research, where tools such as those found in Fig. 3 have made large impacts on research. In materials and chemistry research, tools such as machine learning and deep learning are widely used to analyze complex datasets and make predictions at scales far beyond human capacity.^3,51^ At the same time, advances in natural language processing have led to the development of large language models (LLMs), which have seen widespread adoption both socially and academically for tasks like text generation, information retrieval, and question answering.^52^ Despite their promise, these technologies still face several limitations. Many models operate as powerful pattern-recognition systems, but lack true understanding, contextual awareness, or reasoning abilities akin to those of human scientists. This creates challenges in applying artifical intelligence towards tasks that require scientific intuition, causal inference, or hypothesis generation. As a result, moving from narrow applications toward systems that can contribute meaningfully to autonomous scientific discovery remains a major frontier. Researchers are actively exploring ways to develop artificial intelligence that not only learns from data but also mirrors more complex aspects of scientific reasoning—enabling more creative, robust, and interpretable contributions to research.

Artificial intelligence has already made a significant impact on advancements in chemistry. It has been widely applied in drug discovery, materials development, and property prediction for molecules.^7,53,54^ Natural language processing has also been leveraged in chemistry, such as for predicting activity coefficients directly from SMILES notation.^55^ In analytical chemistry, AI is increasingly combined with spectroscopy and data analysis platforms, with ChemOS serving as one prominent example.^56^ Autonomous laboratories likewise rely heavily on AI to increase the efficiency and accuracy of experiments, aided by platforms like SciFinder and IBM Watson for literature mining and decision support.^57,58^ AI systems have also been applied to reaction prediction and synthesis planning, as seen in tools like Chematica and RXN for Chemistry, which are designed to optimize complex reaction pathways and propose viable synthetic routes.^59,60^

More recently, LLMs have gained significant traction in chemistry-related research and applications across academia and industry.^61–63^ Models such as ChemGPT, ChemBERTa and its successor ChemBERTa-2, and SciBERT have been developed to handle domain-specific tasks more efficiently than general-purpose models, thanks to their training on chemistry-relevant literature and datasets.^13,64–66^ Other examples include ChemCrow, which is used to handle multi-step tasks such as synthesis planning and execution, and ChatMOF, which is designed to handle metal–organic framework (MOF)-specific research.^67,68^ These specialized models have demonstrated improved performance in tasks such as named entity recognition, sentence classification, property prediction, compound generation, and reaction planning. Because many of these tasks have traditionally required manual effort, the use of LLMs presents an opportunity to automate routine work and facilitate the analysis of high-dimensional data, enabling researchers to focus on more complex, creative challenges. While the rapid adoption of LLMs in both educational and professional settings has sparked debate, their impact on literature analysis, knowledge extraction, scientific writing, and laboratory automation is undeniable. As these models continue to evolve, their integration into the research workflow is likely to deepen—further affecting how scientists explore, understand, and communicate chemical knowledge.

Despite the growing success of AI applications in chemistry, several key challenges remain. First, the performance and generalizability of AI models are heavily influenced by the quality and diversity of training data. Many chemical datasets are incomplete, biased, or poorly annotated, which can compromise the accuracy of predictions and limit model applicability across chemical domains. Second, interpretability remains a major hurdle—many models, particularly deep neural networks and large language models, function as “black boxes”, making it difficult for researchers to trust or rationalize their outputs in critical tasks like drug discovery or materials design. Third, while AI can rapidly generate hypotheses or predict outcomes, translating these predictions into experimentally validated results is not always straightforward. The integration of AI into laboratory workflows requires careful alignment between computational insights and experimental constraints. Fourth, the computational cost associated with training and deploying large models can be substantial, creating accessibility barriers for research groups without high-performance computing infrastructure. Finally, the widespread adoption of AI tools raises important ethical and legal considerations, including concerns about data privacy, model accountability, and the proper attribution of intellectual contributions in research driven or assisted by AI. Addressing these limitations is essential to ensure the reliable, equitable, and meaningful integration of AI into chemical science.

Despite the advances made with natural language processing and large language models, there are still significant shortcomings and limitations in what these tools can reliably achieve. A major issue with LLMs is that, on the surface, the generated output often appears correct and is worded in a manner that sounds well-researched. However, a closer inspection—especially in contexts involving complex literature or technical discussion—reveals that the content can be shallow, lacking the substance or nuanced understanding typically demonstrated by domain experts. This stems from the architecture and training processes of these models. LLMs tokenize input text and learn patterns through pretraining to predict the next token (as in GPT models) or masked tokens (as in BERT). While this allows the generation of fluent language, it does not confer actual understanding or reasoning ability. These models are ultimately shaped by the data they are trained on. They cannot interpret unknown terminology, infer abstract meaning, or validate facts in a logical framework. If a model produces coherent, intelligent-sounding responses, it is only mimicking the tone and structure of its training corpus—there is no semantic depth or critical thinking behind the output. Consequently, even highly polished outputs can be misleading or factually incorrect, particularly when the input subject is niche, poorly represented in the training data, or emergent.

As a user, it is hard to notice these deficiencies, especially for non-experts who may not detect subtle errors or oversights. For general queries, LLMs can provide useful summaries or generate ideas. However, in scientific domains like chemistry and materials science, errors regarding LLM-generated content can be particularly dangerous. Reports have shown that overrealiance on LLM-generated results is hazardous and potentially disastrous within laboratory settings.^69^ In particular, misleading descriptions of synthesis routes, incorrect reagent ratios, or false interpretations of chemical safety data can have catastrophic consequences in a laboratory setting. In synthetic chemistry, even minor errors in molecular stoichiometry or handling protocols can lead to failed experiments, wasted resources, or hazardous incidents. Therefore, it is critical that scientists treat LLM-generated suggestions with skepticism and verify all outputs through proper scientific channels before integrating them into experimental designs.

LLMs are also particularly susceptible to echo chambers due to their reliance on quality data. Echo chambers begin to emerge when LLMs train on data that is unbalanced and biased towards particular perspectives, ideologies, or notable voices within the particular area in question. Consequent use of a model trained on such data introduces user bias to the model *via* questions that follow similar trains of thought, further reinforcing certain ideas at the cost of alternative, yet valid perspectives. Citations are also reused and often limited to highly cited papers, creating a gatekeeping environment where newer or lesser-known works are ignored by the model. Lastly, if the machine continues to train and is given output provided by other LLMs or other AI-generated content, then the biases are reinforced exponentially, thus establishing the echo chamber.

Echo chambers encountered with LLMs are problematic because they often remove diversity from their output by excluding alternative, lesser-known sources of work as well as steer away from interdisciplinary perspectives as well as minority voices. Additionally, users run the risk of assuming the model's output is true and fail to investigate the validity of the model's output. The looping encountered with echo chambers also leads to unbalanced visibility and influence the popularity of specific perspectives by giving them greater visibility. Finally, the model is susceptible to collapse where semantically-rich output is lost as it self-loops and retrains on LLM-generated data. This self-reinforcing loop limits the diversity of scientific thought, often ignoring emerging research, interdisciplinary insights, or minority perspectives.

In the context of chemistry and materials science, this lack of diversity and critical reasoning stifles innovation. For a chemical reaction or for material design, for instance, LLMs might preferentially suggest molecular and material derivatives of known compounds rather than truly novel scaffolds. This risks over-optimization within narrow chemical spaces while neglecting vast regions of potentially useful compounds. Similarly, materials science research often involves combining sparse, complex, and multimodal data—ranging from synthesis parameters to spectral signatures—data types that LLMs are not naturally equipped to handle in their current form. As a result, these models may fail to identify subtle structure–property relationships or propose realistic synthesis conditions.

Despite these limitations, LLMs hold great promise in chemistry and materials science if appropriately adapted and integrated. Improvements can be made in several areas. First, dataset curation must go beyond scraping well-known databases—models should be trained on diverse chemical data, including negative results, preprints, patents, and lab notebooks. Including datasets from underrepresented subfields or less-cited sources can help broaden the scope of understanding. Moreover, domain-specific tokenization and the integration of structured data formats such as the Simplified Molecular Input Line Entry System (SMILES), crystallographic information file (CIF), and synthesis trees could enable models to more accurately process chemical information.^70–72^ Another promising direction is to couple LLMs with symbolic reasoning or rule-based logic engines, allowing them to cross-check results with established chemical knowledge.^73,74^ Integration with ontologies and expert systems could help LLMs flag nonsensical reactions, incompatible reagents, or dangerous procedures. For example, a model trained not only on text but also on reaction and materials databases (like Reaxys, PubChem, Materials Project, CADS) and safety protocols (such as the Globally Harmonized System of Classification and Labelling of Chemicals (GHS) or Material Safety Data Sheet (MSDS) data) could serve as a reliable assistant for reaction planning, guiding users while maintaining experimental integrity.^1,75–78^ Beyond serving as writing or synthesis advisors, LLMs could also be integrated with autonomous laboratory platforms. In such systems, the LLM could act as an interface layer: translating natural language instructions into machine-readable formats or suggesting new experiments based on prior outcomes. For this to succeed, the LLM must be tightly coupled with reinforcement learning agents, electronic lab notebooks, robotic control systems, and real-time analytics tools like spectroscopy or chromatography instruments. The model would also need access to structured experimental databases and semantic representations of chemical knowledge, enabling it to reason beyond language.

## 4 Integrating AI and robotics: the next generation of autonomous laboratories

Automation and artificial intelligence, in combination with high-throughput experimental approaches, are rapidly becoming cornerstone technologies in scientific research.^79–81^ Just as the invention of early instrumentation such as the scanning electron microscope (SEM) initially comes with prohibitively high costs and manufacturing limitations, so too does the current wave of laboratory robotics face similar barriers. Over time, SEMs are optimized, standardized, and mass-produced—democratizing access across research institutions. A parallel transformation is beginning to unfold for autonomous laboratories. A future is anticipated where robotics—much like past innovations—becomes affordable, modular, and widely adopted, paving the way for an era of accessible, AI-driven science.

### 4.1 The challenges of integration

**Fig. 4:**
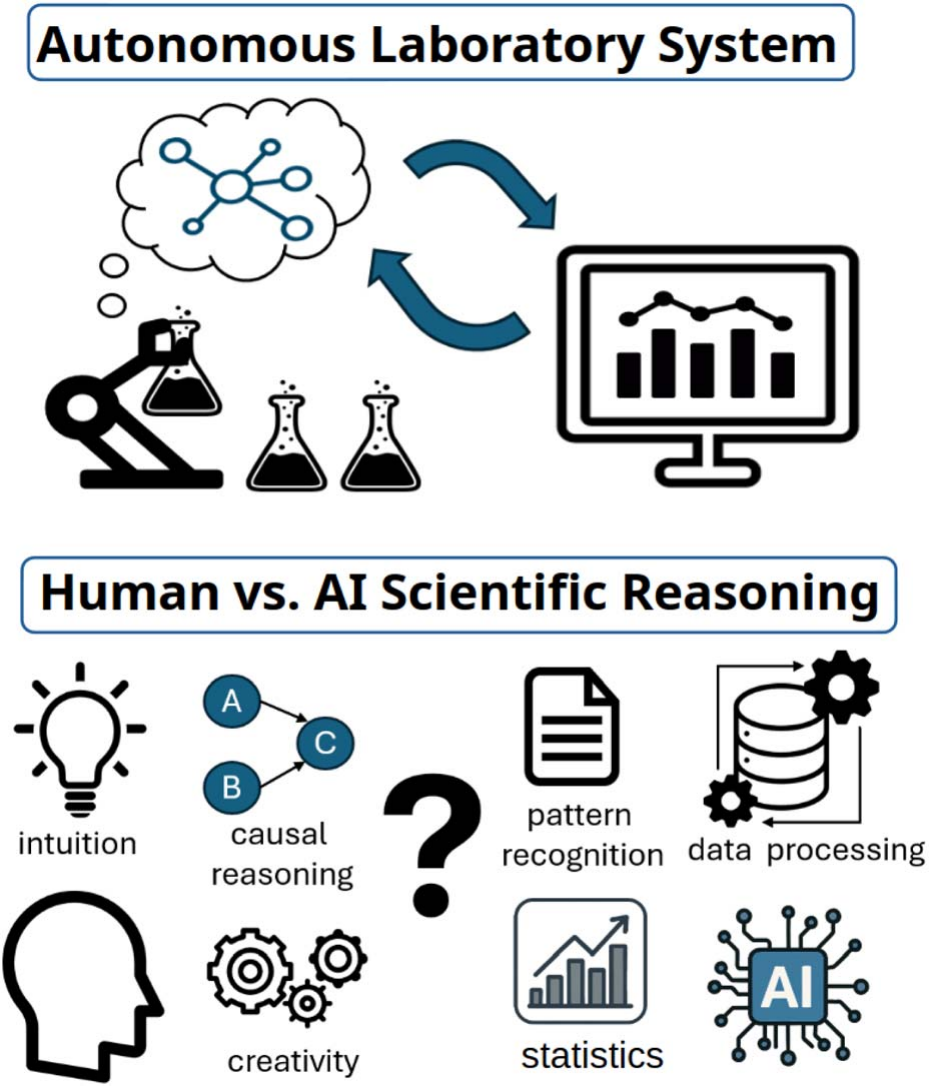
Autonomous laboratory system and its relationship with human *vs.* AI scientific reasoning.

Despite the promise, integrating robotics and AI into current lab environments presents a host of practical challenges. First and foremost is cost. Commercially available laboratory robots range from hundreds of thousands to millions of dollars, effectively restricting their adoption to elite institutions or industry labs with generous funding. Smaller groups—often hubs of creativity and unconventional ideas—are left behind. This financial wall shuts out smaller groups that may otherwise highly benefit from adopting robotics within their labor setups. Beyond cost, environment compatibility presents a major hurdle. In particular, these differences become clearer when comparing the needs of human researchers against the needs of machines and robotics (Fig. 4). Today's labs are designed around the capabilities and limitations of human researchers. Benchtops, hoods, equipment spacing, and interfaces are all optimized for human interaction. Robots, however, operate differently. They don't need eye-level displays, standing desks, or human-scale workspaces to carry out the same tasks that a person might have traditionally been responsible for. A shift toward robot-centric lab design—such as placing equipment in reachable zones for robotic arms, standardizing interfaces, and enabling robotic mobility—could unlock far more efficient automation. Safety is another critical concern. Unlike human researchers, robots can operate for long hours without fatigue whereas researchers experience fatigue and reduced capacities for sound judgement when working over long periods of time with limited rest.^82^ However, this comes with risk. Autonomous labs handling hazardous chemicals must be equipped with robust safety systems- gas sensors, fire detectors, emergency shutoff mechanisms, and protocols, for example- to halt robotic activity during malfunction. Without such systems, a malfunction could escalate into catastrophic damage—a concept it might be called the “robot rampage” scenario.

### 4.2 Open source as an Equalizer

As the race to adopt robotics and automate chemical laboratories escalates, there is a large risk of losing the creativity and unique voices of smaller groups in favor of the fewer, better-funded organizations. Here, open source is a very attractive alternative to consider. Open source refers to when source code is made to be free to use, modify, and distribute. It is community-driven and receives the support of many communities, including developers, who participate in projects by working on debugging efforts, developing features, or improving performance. Open-source hardware and software provide a compelling path toward more equitable access to automation. Just as open-source software like Linux reshaped computing and creativity, open hardware and 3D printing can do the same for science. With tools like Blender, FreeCAD, and OpenSCAD for part design, and 3D printers for fabrication, research groups can build customized robotic systems at a fraction of the cost of commercial products.^83–85^ In this space, community-driven projects like LEGOLAS, SyringeBot, and the FLUID platform are already showing what's possible.^33,86,87^ These systems, often based on modular, open-source electronics like Arduino or Raspberry Pi, allow researchers to retain full control over their experimental workflows while contributing to a shared ecosystem. Just as importantly, open-source designs can be iterated and improved upon by anyone, accelerating progress across the board.

### 4.3 Validating autonomous experiments

While robots can execute tasks tirelessly, experimental validation remains a critical bottleneck. Unlike human researchers, who intuitively adjust based on context—changing pipette angles, modifying stirring speeds, or adding unexpected steps—robots require detailed, pre-defined protocols. When unexpected outcomes occur, machines struggle to adapt or potentially fail altogether. This makes validation protocols essential. Systems must be developed not only to confirm that experiments were executed correctly, but that the results are meaningful and scientifically sound. Human oversight—particularly in comparing outcomes with literature benchmarks or recognizing anomalies—remains indispensable.

### 4.4 Rethinking the human-AI-robot interface

The integration of AI into robotic systems extends beyond motion control or task automation. Developments in Bayesian optimization, reinforcement learning, and active learning now allow robots to optimize experimental conditions in real-time.^88^ But the future of AI in labs lies not only in optimization—it also lies in curiosity-driven exploration. Autonomous systems are envisioned to generate hypotheses, identify unexplored chemical spaces, and iteratively refine their understanding through trial and error—much like a human scientist. Such systems will rely on a fusion of machine learning, large language models (LLMs), symbolic reasoning, and even natural language interfaces. Imagine a robot reading recent papers, extracting reaction trends, designing a new catalytic route, and testing it overnight—entirely autonomously. These systems could learn “unspoken rules” that researchers pick up over years of trial and error. Integration with lab knowledge graphs, reaction ontologies, and semantic search engines will further accelerate this evolution.

### 4.5 Where do we draw the line?

Automation and artificial intelligence are rapidly becoming staple technologies in our research environments. Researchers are now faced with a series of growing pains as they try to streamline these technologies into their lab set-ups and research environments. Incompatibilities between the environments that automation and AI require and the environments that are currently designed for human researchers make it difficult to truly take advantage of the potential that automation and AI offers. Robot-centric design, therefore, should be included when considering research environment design, as such spaces open the possibility of fully automated workflows. Concurrent developments such as sensor-based real-time recognition, inclusion of semantics and meaning during AI-led analysis, and fully autonomous Bayesian optimization and experimental planning all help promote existing AI and automation systems towards true AI-driven autonomy.

As AI-driven autonomy advances, fundamental questions begin to emerge.

• *Should AI be allowed to make independent research decisions?*

• *What are the limits of human-AI collaboration?*

• *Or is there a third path—true partnership, where humans and AI co-create knowledge?*

Autonomous labs have the potential to accelerate discovery dramatically, but they also challenge the traditional roles of researchers. Science is not merely the execution of tasks—it's a creative, interpretive process that thrives on intuition, inspiration, and sometimes serendipity. Can AI truly replicate that? Or will human researchers always serve as the interpreters and guardians of scientific insight? Rather than seeking to replace human intellect, the goal shifts toward symbiosis. Researchers may take the lead in high-level design, interpretation, and imagination, while AI and robotics handle complexity and repetition. In a more integrated model, AI could contribute novel insights or challenge assumptions, prompting new directions in human thought. Such collaborative intelligence may not only change how research is performed—it may redefine what it means to “do research” in the 21st century.

## 5 Future directions and ethical considerations

In time, automation and AI will play a large role in many research groups, forcing researchers to reconsider and possibly redefine the role of scientists in scientific research. As these technologies become normalized, it is likely that the average research experience will shift from typical hands-on experimentation towards AI-guided hypothesis generation and validation. Likewise, as these technologies become widely adopted, a series of new ethical concerns will also arise. Issues such as data bias and its effects on AI-driven material discovery as well as over-reliance on AI-generated insights must be considered. Additionally, the potential loss of human expertise in experimental chemistry is also a growing concern, particularly as generations change and skillsets become lost. Finally, the growing need for interdisciplinary collaboration between chemists, data scientists, and engineers must also be considered, especially when considering data quality and the variety of needs between each group.

## 6 Conclusion: the future of AI and robotics in chemistry

As robotics and AI continue to reshape chemistry and materials research, the scientific community stands on the brink of a transformative era. The integration of these technologies is not just a matter of efficiency or productivity—it represents a fundamental shift in how scientific inquiry is conducted. Traditional human-centric laboratories, once optimized for manual workflows, must now evolve into hybrid spaces where humans and machines operate side by side. This reimagining includes not only physical infrastructure—such as benchtop accessibility, robotic mobility, and interface standardization—but also a cultural and methodological shift toward machine-readable protocols, real-time data pipelines, and AI-integrated decision-making.

Open-source hardware, modular platforms, and digital fabrication tools like 3D printers are already democratizing access to automation. These technologies offer an opportunity to expand accessibility and give smaller or resource-limited labs the ability to participate in the automation revolution. Rather than becoming limited to well-funded institutions, the tools of AI and robotics can be distributed across the global research community, fostering broader innovation and inclusivity. Collaborative, community-driven projects serve as a proving ground for what decentralized scientific progress can look like. At the same time, future development of safety protocols, robust validation systems, and clear experimental frameworks are necessary to ensure reliability and prevent unintended behavior during autonomous operation.

The future of autonomous science is not just technical—it is also ethical, philosophical, and deeply human. The ability of AI to autonomously generate hypotheses, optimize experiments, and interpret results forces one to redefine the very nature of scientific creativity. Where does human judgment end and machine insight begin? Can machines ever truly emulate the serendipity, intuition, or conceptual leaps that characterize human discovery? While current systems support optimization and planning, the next generation aims to navigate unexplored chemical spaces and learn from data like human researchers. This evolution raises a critical question: Should AI make independent research decisions, or should it remain a tool guided by human insight? A third path—collaborative intelligence—offers a compelling future, where humans focus on creativity and interpretation while AI handles complexity and exploration.

To fully realize the potential of automonomous laboratories, future laboratories will require not only technological innovation but also thoughtful systems for validation, safety, ethical governance, and interdisciplinary collaboration. Chemists, engineers, data scientists, and ethicists must work in concert to build research environments that are not only intelligent but also transparent, robust, and trustworthy. Together, these systems could amplify scientific creativity, accelerating discovery while preserving the human essence of inquiry. Ultimately, the integration of AI and robotics is not just about how we conduct research—it is about rethinking what it means to do science in the 21st century. As we step into this new paradigm, the challenge is not simply to build smarter tools, but to build smarter systems—ones that elevate human insight rather than replace it, and that expand the frontiers of knowledge while remaining grounded in the values that define scientific progress.

## Author contributions

All authors have contributed towards conceptualization, writing – original draft, and writing – review & editing. Funding acquisition: L. T. and K. T.

## Conflicts of interest

There are no conflicts to declare.

## Data Availability

No primary research results, software or code have been included and no new data were generated or analysed as part of this review.
